# Combined Stabilizing of the Solid–Electrolyte Interphase with Suppression of Graphite Exfoliation via Additive-Solvent Optimization in Li-Ion Batteries

**DOI:** 10.1021/acsami.3c10792

**Published:** 2023-10-18

**Authors:** Sanghamitra Moharana, Geoff West, Ashok S. Menon, Wilgner Lima da Silva, Marc Walker, Melanie J. Loveridge

**Affiliations:** † Warwick Manufacturing Group (WMG), University of Warwick, Coventry CV4 7AL, U.K.; ‡ Department of Chemistry, University of Warwick, Coventry CV4 7AL, U.K.; § Department of Physics, University of Warwick, Coventry CV4 7AL, U.K.

**Keywords:** propylene carbonate, graphite exfoliation, Li dendrites, potassium cation, solid electrolyte interphase, lithium fluoride, lithium-ion batteries

## Abstract

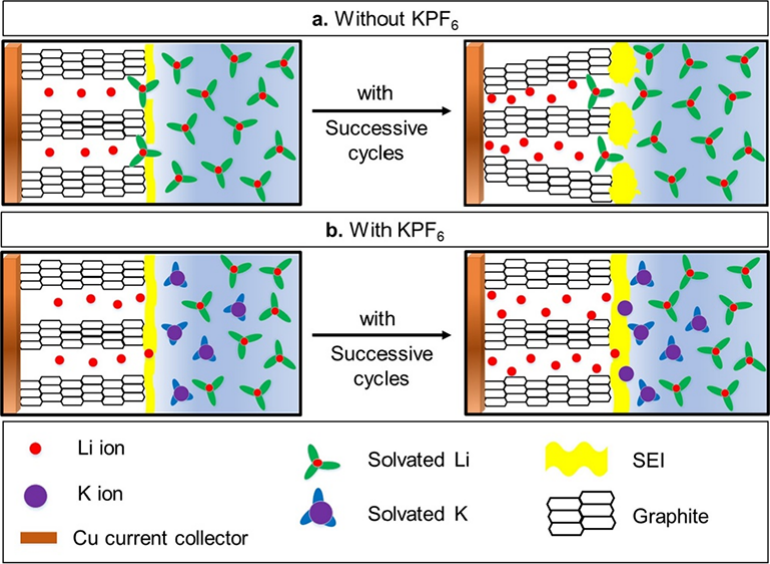

Propylene carbonate (PC) is a promising solvent for extending the operating temperature range for lithium-ion batteries (LIBs) because of its high dielectric constant and wide temperature range stability. However, PC can cause graphite exfoliation through cointercalation, leading to electrolyte decomposition and subsequent irreversible capacity loss. This work reports the formulation of a ternary electrolyte with the introduction of an inorganic salt additive, potassium hexafluorophosphate (KPF_6_), to address the aforementioned concerns. We demonstrate the cumulative effect of solvent and additive on delivering multiple performance benefits and safety of the battery. The faster diffusion rate of K ^+^ solvation shell decreases the rate of PC decomposition, thereby reducing its cointercalation. Additionally, the optimum concentration of KPF_6_, i.e., 0.1 M constructs a robust and insoluble LiF-rich electrode/electrolyte interphase, further suppressing graphite exfoliation and Li dendrite formation. The stable cyclability is achieved by enhanced Li ^+^ transportation through the LiF-rich interphase, enabling an exfoliation-free and dendrite-free graphite anode in the ternary electrolyte.

## 1 Introduction

Lithium-ion batteries (LIBs) have achieved great success in portable electronics and are an established technology for energy storage systems since their original commercialization in 1991.^1^ The performance of LIBs is dependent on many internal and external factors. One of these key factors is the operating temperature of LIBs, which is limited by the temperature range of the electrolyte (stability window), affecting its inherent properties such as ionic conductivity, viscosity, and charge-transfer resistance.^2−4^ Therefore, in order to expand the operating temperature range of a LIB, the electrolyte’s temperature range is often modified by incorporating solvents and/or additives. One of the electrolyte solvents, propylene carbonate (PC) has a wide temperature range, i.e., −48.8 to 242 °C, which makes it a desirable organic solvent for more extreme temperature operations.^5,6^ However, the exfoliation of graphite layers caused by PC cointercalation results in electrolyte decomposition, which gives rise to concerns for present-day LIB.^7−10^

Liquid electrolytes comprising solvents, a Li salt, and additives act as a medium for reversible Li ^+^ ion transportation between the electrodes in conventional LIBs. Usually, a mixture of cyclic carbonates is combined with linear carbonates in various proportions to produce an electrolyte solvent with suitable viscosity and ionic conductivity. Although the high dielectric constants of cyclic carbonates (ethylene carbonate (EC) and PC) promote dissolution and dissociation of Li salt, the high viscosity leads to their low ionic conductivity.^11^ Therefore, less viscous linear carbonates (dimethyl carbonate (DMC), ethyl methyl carbonate (EMC), and diethyl carbonate (DEC)) are added to enhance the Li ^+^ ionic conductivity of the electrolyte.^12^ Among all the electrolyte solvents, EC is irreplaceable as it becomes reduced first on the anode surface^11,13,14^ forming a flexible electrode/electrolyte interphase–solid electrolyte interphase (SEI).^15^ However, EC has a high melting point (36.4 °C), hence staying in the solid state at room temperature. Therefore, EC is more likely to crystallize even in the presence of linear carbonate (when temperature decreases), resulting in increased viscosity and charge-transfer resistance. This phenomenon is more pronounced especially when the battery is operated at temperatures closer to subzero °C, thus limiting the working temperature range.^4,16^ Therefore, one of the approaches is to incorporate PC as a third solvent in order to form a ternary electrolyte, which reduces the liquidus temperature (hence suppressing EC crystallization), leading to possible improvements in both the ionic conductivity and the cyclability of the cell compared to the binary electrolyte system.^17−19^

PC is a suitable solvent for battery electrolytes as it has structural similarities with EC (extra methyl group attached to ring) in addition to the wide temperature range.^8^ Furthermore, the high dielectric constant of PC facilitates the dissociation of salts in the electrolyte. However, graphite anodes are not compatible with PC, as it is unable to stabilize the SEI film on the anode surface.^7,8^ Subsequently, PC cointercalates into the graphite layers with Li ^+^, leading to its exfoliation. Additionally, this loosely bound SEI film does not block electrons efficiently, resulting in the reduction of electrolyte and Li ^+^, thereby promoting electrolyte decomposition and Li metal deposition, respectively, even at low charging rates.^11,15^ These phenomena consume active Li through electrolyte decomposition, promoting SEI damage and delamination and thereby decreasing the reversible capacity of the cell.

A stable SEI construction is essential to suppress graphite exfoliation in order to maintain the cyclability of the cell.^20^ In this regard, the most effective approach is to introduce additives into the electrolytes for building an effective SEI film on graphite anodes with PC-based electrolytes. Recently, it has been demonstrated that the incorporation of a small amount of salt additives can suppress PC decomposition on graphite materials by improving the performance of the cell.^21−24^ Similarly, Chandrasiri et al. discovered that both cesium and potassium acetate result in thinner SEI layers in PC-based electrolytes compared to other alkali acetates.^22^ Numerous studies have been conducted further on salt additives in PC-rich electrolytes and have been found to have significant performance enhancement with graphite anodes.^6,22−24^ For instance, the CsPF_6_ additive suppresses graphite exfoliation in PC-rich electrolytes by producing an ultrathin and compact SEI film on its surface.^23,24^ In addition, this modified electrolyte provides excellent wide-temperature performance due to the extended temperature stability of the PC solvent.^6,19^ Furthermore, CsPF_6_ as an electrolyte additive efficiently inhibits Li dendrite growth enabling improved Li ^+^ transportation through the SEI layer.^23,25−28^ However, potassium electrolyte additives have not been thoroughly investigated in PC-containing electrolytes with regard to impacting both graphite exfoliation and Li dendrite formation, thus not demonstrating the multiple PC solvent-potassium additive correlations on graphite. Moreover, the impact of additive concentration along with PC solvent has not yet been fully explored. Therefore, this study is conducted to establish the understanding of the electrolyte solvent, additive, and its combined effect on the inhibition of Li dendrite formation and growth and graphite exfoliation in order to enhance the cell’s performance.

In this work, a potassium additive, potassium hexafluorophosphate (KPF_6_), is systematically investigated with PC solvent on graphite in order to evaluate the impact of the additive and its underlying mechanism. The primary interest of this comprehensive study is to formulate an optimized electrolyte by investigating the influence of additive concentrations and volume proportions of PC and EC solvents for suppressing graphite exfoliation and Li dendrite formation/growth. Ultimately, this will deliver a superior electrochemical performance and expand the lifetime of the battery.

## 2 Experimental Procedure

### 2.1 Materials

Single-side-coated artificial graphite (Hitachi MagE3) and LiNi_0.6_Mn_0.2_Co_0.2_O_2_ (Targray NMC 622) electrodes were provided by the CAMP Facility, Argonne National Laboratory, USA. The details of the electrodes are stated in Table S4. The commercial electrolyte consisting EC:EMC (3:7 v/v), 1 M lithium hexafluorophosphate (LiPF_6_), and 1 wt % VC (vinylene carbonate) named as RD281 was used in this work (PuriEL, Soulbrain). Battery grade EC ( ≥ 99 %, acid <10 ppm, H_2_O < 10 ppm), PC ( ≥ 99 %, acid <10 ppm, H_2_O < 10 ppm), and EMC ( ≥ 99.9 %, acid <10 ppm, H_2_O < 10 ppm) solvents along with LiPF_6_ (battery grade ≥99.99 % trace metal basis) and KPF_6_ ( ≥ 99 % ) were purchased from Sigma-Aldrich for formulation of electrolytes. Both Li and K salts were dried under a vacuum to remove the excess moisture. The formulation of electrolytes was carried out inside an argon-filled Mbraun glovebox (O_2_ and H_2_O < 0.5 ppm). The concentrations of EMC, LiPF_6_, and VC additive are kept constant in order to avoid its influence on the electrochemical performance of the cell. This implies the volume percentage of PC varies with the volume percentage EC and vice versa.

### 2.2 Electrochemical Testing

Graphite and NMC 622 electrodes were vacuum-dried overnight at 50 °C before use. These electrodes were cut into 15 and 14.8 mm Ø and 15 mm Ø, respectively, and assembled into Hohsen 2032-type coin cells inside the glovebox using a Celgard—2325 separator (19 mm Ø) and modified electrolytes of 100 μL. Graphite | NMC 622 full cells (N/P ratio = 1.2) cycling was carried out in constant current-constant voltage (CCCV) charging and constant current (CC) discharging mode in 3–4.2 V voltage range. Two formation cycles were employed at C/20 (∼0.14 mA), followed by cycling at C/5 (∼0.55 mA) to 100 cycles at room temperature. The electrochemical properties are evaluated on a BTS (Battery Technology Source) battery tester.

ECC-PAT-core three-electrode cells were used for monitoring the potential of each electrode distinctively with respect to the reference electrode. Graphite and NMC 622 electrodes were cut into 18 mm Ø discs and assembled into an EL-cell type using NMC 622 as a working electrode (WE), graphite as a counter electrode (CE), and inbuilt Li ring as a reference electrode (RE) in the insulation sleeve. The insulation sleeve had an inbuilt Whatman Borosilicate glass fiber separator of 260 μL thickness. EL-cells were cycled in CC mode in the 3–4.2 V voltage range.

Electrochemical impedance spectroscopy (EIS) was measured using a three-electrode EL-cell at 50 % SoC (state-of-charge) in ambient temperature. EIS measurements were taken place after the first cycle and every 10 cycles until 100 cycles. All the measurements were conducted in VMP3 potentiostat (Bio-Logic) in the frequency range of 500 kHz–10 mHz with a voltage amplitude of 10 mV. The cells were held for 30 min of relaxation time to achieve the equilibrium state prior to EIS measurements. All of the EIS spectra were fitted with a simplified Randles circuit using Zview software.

### 2.3 Postmortem Characterization

The coin cells were disassembled after 100 cycles under fully discharged conditions (3 V) inside the glovebox to reduce moisture contamination. Afterward, an airless transfer device was used to transfer the cycled graphite anodes from the glovebox to the equipment chamber for postmortem characterization. The cycled electrodes are cleaned carefully with dimethyl carbonate (DMC) to remove the excess electrolyte salt deposits before transferring the samples to the equipment chamber.

Scanning electron microscopy (SEM) was performed to investigate the morphological evolution of the cycled graphite anodes. The SEM images were recorded in a Zeiss Sigma field-emission SEM (FM-SEM) (Sigma, Zeiss) equipped with an energy-dispersive X-ray spectrometer (EDX). The images were taken with an accelerating voltage of 10 kV and an aperture size of 60 μm. In-lens detection was used to collect the SEM images. EDX was performed to determine the elemental composition of the potassium dendrites.

X-ray diffraction (XRD) was conducted in a Malvern PANalytical Empyrean diffractometer with nonmonochromatic Cu Kα radiation, using a PiXcel3D detector operating in 1D scanning mode. Data were collected in Bragg–Brentano (reflection) mode from 5° to 47° (2θ) with 0.013° step size and scan speed of 0.011°/s. Afterward, the XRD peaks were analyzed by the Pawley refinement^29^ method using Topas Academic (v6) software.^30^ Since the measurements were taken in reflection mode, the samples could be displaced to certain degrees in the direction normal to the sample plane, which introduced height error. To address this height error, first, the reflection from the copper current collector was fitted for each sample using a cubic (*Fm*3̅*m*) unit cell with fixed lattice parameter 3.615 Å,^31^ with a refinement in sample displacement (SD). This method is reasonable, as the Cu current collector does not participate in the electrochemical redox reactions. Following this, the *d*-spacing and peak width (full-width at half-maximum (fwhm)) of graphite was obtained by fitting the 002 reflection with a hexagonal (*P*63/*mmc*) unit cell, with the refinement in its unit cell parameters (a and c). In this process, the SD obtained from Cu 111 reflection was used for fitting graphite 002 reflection (important to note: SD is not refined again for fitting graphite 002 reflection). In all refinements, a third-degree Chebychev polynomial background function was used. The peak shapes were modeled using a pseudo-Voigt function combined with an additional function to account for the peak asymmetry (due to the axial divergence of the X-ray beam).

X-ray photoelectron spectroscopy (XPS) measurements were performed in a Kratos Axis Ultra DLD spectrometer (Kratos Analytical Ltd.) with a monochromatic Al Kα X-ray (1486.7 eV) source for excitation. The work function of the spectrometer was calibrated using Fermi edge and 3d_5/2_ peak, recorded from a polycrystalline Ag sample. XP spectra were recorded at a takeoff angle of 90° and at a pass energy of 20 eV (resolution ∼0.4 eV). In order to negate surface charging effects, the surface was flooded with a beam of low-energy electrons during the experiment. The recorded binding energies were recalibrated by referencing the C – C/C–H component of the C 1s spectra to 284.8 eV during fitting using the CasaXPS software package. The data were fitted using Shirley backgrounds and mixed Gaussian–Lorentzian (Voigt) lineshapes.

SIMS experiments were performed in an FEI Scios dual-beam scanning electron/focused ion beam (FIB) microscope equipped with a Quadrupole Mass Analyzer (QMA). High vacuum conditions below 5 × 10 ^–6^ mbar were maintained for conducting SIMS experiments to avoid the collision of ejected secondary ion species with the background gas molecules. Both positive and negative ion mode mass spectra were obtained by sputtering Ga ^+^ source ions on the graphite anodes at an accelerating voltage of 30 kV and beam current of 0.5 nA. MASsoft Professional 7 software was used for recording the mass spectra data. SIMS mapping was recorded with ion beam of 10 pA using a Hiden Analytical SIMS mapper installed in Scios FIB/SEM. The mapping images were acquired using the acquisition time set to 0.6 ms (dwell time of 0.4 ms and settle time of 0.2 ms) with 0.01 increment.

## 3 Result and Discussion

### 3.1 Electrochemical Testing

**Figure 1:**
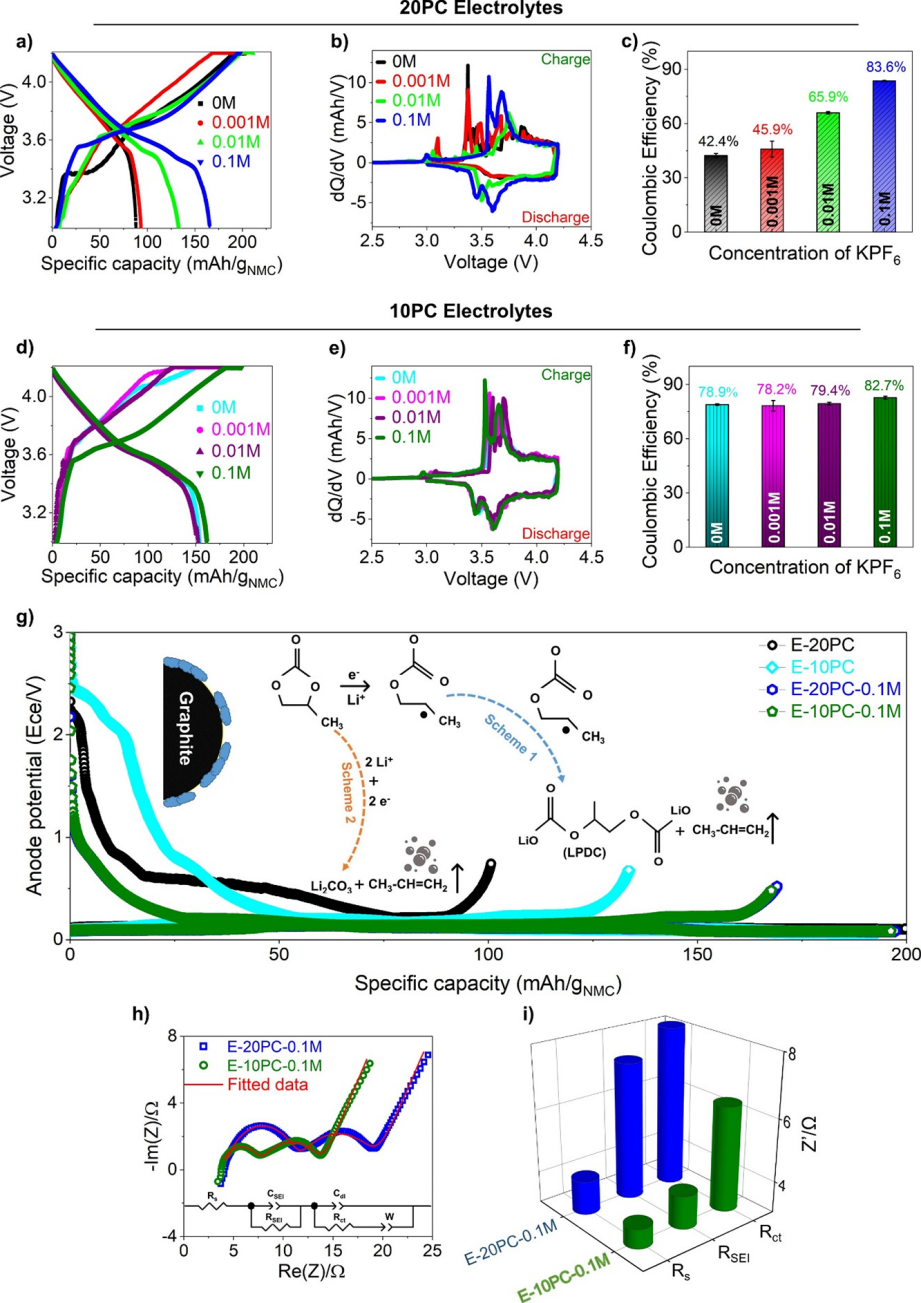
Electrochemical performances of NMC 622|graphite cell using different electrolytes during its formation cycle. (a, d) Voltage vs specific capacity, (b, e) incremental capacity plot, (c, f) Coulombic efficiency of 20 and 10 vol % PC electrolytes with various additive concentrations (0 – 0.1 M), respectively. (g) Three-electrode anode potential profile and reaction schemes of PC solvent. (h) EIS at 50 % SoC after the formation of 0.1 M KPF_6_ in 20 and 10 vol % PC, equivalent circuit for EIS fitting (series resistance; *R*_S_, SEI layer resistance; *R*_SEI_, charge-transfer resistance; *R*_ct_, constant phase element for SEI and double layer; *C*_SEI_ and *C*_dl_, Warburg impedance; W). (i) Comparison of EIS fitting results for different electrolytes after formation.

Graphite | NMC 622 full cells were cycled with modified electrolytes with various concentrations of KPF_6_ and volume percentages of PC and EC, as listed in Table S1. The formation cycle voltage profile is presented in Figure 1a with 20 vol % PC without KPF_6_ additive (20PC, 0M; E-20PC electrolyte). An unusual slope change in voltage profile at ∼3.37 V (Figure 1a, black colored plot) implies the excessive electrolyte decomposition during charging. This is clearly evident as crowded peaks which can be seen in the incremental capacity plot (d*Q*/d*V* vs *V*) in Figure 1b (separately presented in Figure S1a), delivering a low specific discharge capacity of ∼88 mA h/g and Coulombic efficiency (CE) of 42.4 % (Figure 1c). This indicates the solvated Li ^+^ intercalation and continuous electrolyte decomposition through active Li ^+^ ion consumption at the graphite edges as the charging of the cell proceeds. The available fresh active material decomposes the E-20PC electrolyte because of the unstable and loose SEI layer, providing a notion of graphite exfoliation through PC cointercalation.

Furthermore, the reduction in the volume percentage to 10 vol % PC shows increased capacity of ∼154 mA h/g and CE of ∼78.9 % (without KPF_6_; E-10PC) in Figure 1d,f. The incremental capacity peaks in Figure 1e (Figure S1b) demonstrate that the electrolyte decomposition is diminished in E-10PC compared to E-20PC. This establishes that the SEI layer produced on the graphite anode in 10 vol % PC electrolytes is more efficient in restricting electrolyte decomposition compared to 20 vol % PC-based electrolytes. However, relatively high polarization is observed in the E-10PC voltage profile, indicating low charge acceptance of graphite (Figure 1d). A significant enhancement in electrochemical performance is noticed in both 10 and 20 vol % PC electrolytes with an increase in KPF_6_ additive concentration.

Ultimately, 0.1 M KPF_6_ in the 20PC electrolyte escalates the specific discharge capacity to ∼159 mA h/g and CE of 83.6 %, presenting sharp and distinct d*Q*/d*V* vs *V* peaks during charging/discharging. The peak at ∼2.9 V is attributed to the electrolyte decomposition peak, which is not present afterward during the charging of the cell, indicating the inhibition of electrolyte decomposition. The oxidation peaks at ∼3.65 and ∼3.75 V are attributed to Li ^+^ intercalation into graphite, NMC phase transition from hexagonal to monoclinic phase (H1 → M), respectively, with the corresponding reduction peaks during discharge.^32^ Similarly, the polarization is decreased in the E-10PC-0.1 M electrolyte, achieving discharge capacity of ∼161 mA h/g and CE of ∼82.7 % . The shifting of the oxidation peaks in the incremental capacity plot (d*Q*/d*V* vs *V*) toward the left and reduction peak toward the right direction indicates that the polarization is reduced with the addition of 0.1 M KPF_6_ into 10 vol % PC electrolyte. For instance, the first oxidation peak at ∼3.57 V in E-10PC (attributed to Li ^+^ intercalation into graphite; cyan-colored plot) shifts to ∼3.5 V with the addition of 0.1 M KPF_6_ (green-colored plot), indicating reduced polarization in E-10PC-0.1 M electrolyte. The reproducible cycling data and CE are presented in parts S2 and S3.

The graphite potential observed in the three-electrode system shown in Figure 1g confirms the extensive electrolyte decomposition in both E-10PC and E-20PC, resulting in lower discharge capacities. The schematic diagram presents the formation of electrolyte decomposition products, e.g., lithium propylene dicarbonate (LPDC) and Li_2_CO_3_ through the ring opening of PC during the formation cycle. This SEI constituent, e.g., LPDC is less cohesive while depositing on the graphite surface, and more soluble compared to lithium ethylene dicarbonate (LEDC), the decomposition product of EC. It is, therefore, unable to form a solid and compact SEI layer.^8^ Additionally, propene gas (CH_3_ – CH=CH_2_), which is a byproduct in both schemes 1 and 2 in Figure 1g, generates microcracks in the particles, enabling fresh graphite for electrolyte decomposition.^10^ Nevertheless, the addition of 0.1 M KPF_6_ in both 10 and 20 vol % PC significantly enhances the capacity of the cell by suppressing electrolyte decomposition (Figure 1g). EIS results are shown in the Nyquist plot in Figure 1h, demonstrating relatively high series resistance (*R*_s_), SEI resistance (*R*_SEI_), and charge-transfer resistance (*R*_ct_) for E-20PC-0.1 M (Figure 1i), implying that E-10PC-0.1 M is the optimum electrolyte.

**Figure 2:**
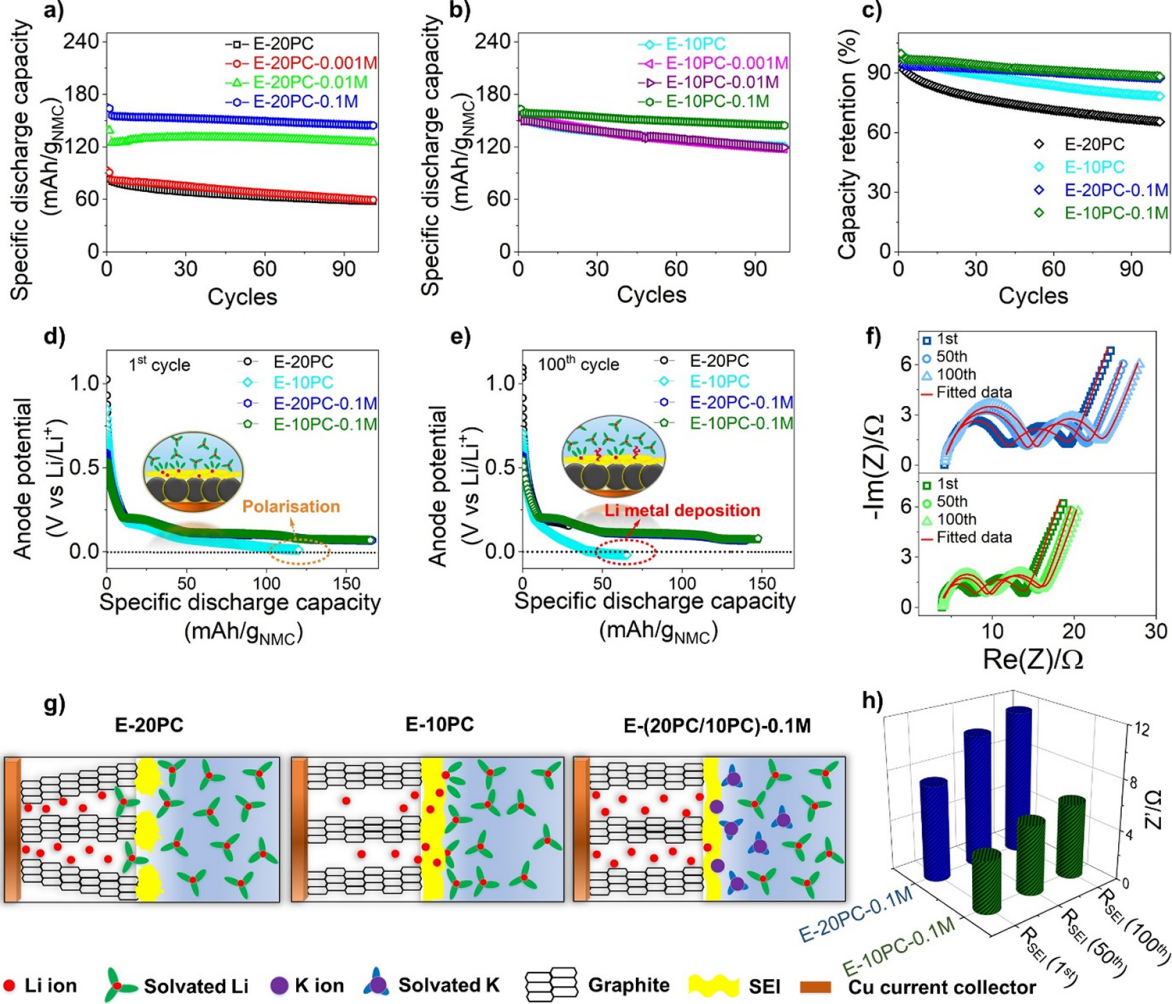
Electrochemical performances of NMC 622|graphite cell using different electrolytes during its cycling. Cycling stability of (a) 10 vol % PC and (b) 20 vol % PC with different concentrations of additive, (c) capacity retention comparison of with and without additive, (d) 1st cycle, and (e) 100th cycle anode potential comparison of the cells cycled with the electrolytes with optimum and without additive, (f) EIS at 50 % SoC after 1st, 50th, and 100th cycles with E-20PC-0.1 and E-10PC-0.1 M electrolytes with fitted results, (g) schematic diagram of plausible mechanism behind the performance of the cells cycled with E-10PC, E-20PC, E-20PC-0.1, and E-10PC-0.1 M, and (h) comparison of *R*_SEI_ of E-20PC-0.1 and E-10PC-0.1 M electrolytes after 1st, 50th, and 100th cycles (obtained from equivalent circuit fitting of Nyquist plots presented in Figure 2f).

Figure 2a,b shows a declining trend in discharge capacity for both 10 and 20 vol % PC electrolytes without additives. It should be emphasized that the discharge capacity is almost doubled by reducing the PC content to half (10 vol %), implying the increment is due to PC content only (Figure S4). Lower PC content produces less LPDC (more LEDC from higher EC) and propene gas and hence declines the probability of graphite exfoliation through cointercalation. This phenomenon produces a relatively stable SEI on the graphite surface, ensuring longer-term cyclability. However, the graphite potential measured from three-electrode cells indicates a developed polarization in the first cycle (Figure 2d) for E-10PC, leading to Li deposition at the 100th cycle (Figure 2e). The increase in the polarization in E-10PC electrolytes is due to the high viscosity of PC solvent, which hampers Li ^+^ transportation into the anode.^11^ The solid-state diffusion of Li ^+^ into graphite is hampered by the developed polarization, leading to the overpotential of anode (also shown in Figure 1d) during the charging of the cell. This increased overpotential in Figure 2d eventually causes metallic Li deposition through Li ^+^ to Li^0^ reduction, as shown in Figure 2e.

Moreover, the graphite voltage profile is maintained with an optimum 0.1 M concentration delivering superior discharge capacity by suppressing possible exfoliation and Li deposition (Figure 2d,e) for 20 and 10 vol % PC, respectively. 0.1 M KPF_6_ maintains the cyclability (Figure 2a,b,e) and capacity retention (Figure 2c) of the cell, irrespective of the PC content. A schematic diagram in Figure 2g proposes a possible mechanism of the KPF_6_ additive influence in PC-based electrolytes, where a more stable SEI is predicted with 0.1 M KPF_6_-explained elaborately as follows.

High PC content in E-20PC increases the probability of soluble electrolyte decomposition products for SEI formation and therefore fails to construct a stable SEI film on the graphite surface. This leads to graphite exfoliation by PC cointercalation, exposing the active graphite edges to the electrolyte, and promoting excessive electrolyte decomposition. This phenomenon consumes available Li ^+^ inventory for continuous SEI formation and growth upon cycling, exhibiting the inferior performance of the cell (presented in the first image in Figure 2g).

In E-10PC, less PC content decreases the probability of graphite exfoliation via PC cointercalation by building a comparatively stable SEI layer; however, the high viscosity of PC solvent increases polarization by inhibiting faster Li ^+^ transportation to graphite anode. As discussed previously, this develops the overpotential of the anode, which results in a gradual capacity decline upon cycling (presented in the second image in Figure 2g).

For optimum additive concentration (0.1 M), a compact SEI film is produced on the graphite anode, which suppresses electrolyte decomposition and polarization developed in E-20PC and E-10PC, respectively. This demonstrates the superior performance of the cells cycled with E-20PC-0.1 M and E-10PC-0.1 M electrolytes (presented in the third image in Figure 2g). Moreover, E-10PC-0.1 M proves to be the optimized electrolyte because of its low *R*_SEI_ throughout cycling compared to E-20PC-0.1 M (Figure 2h), evaluated from the EIS study in Figure 2f.

### 3.2 Postmortem Characterization: Combined Suppression of Graphite Exfoliation and Li Dendrite Growth

**Figure 3:**
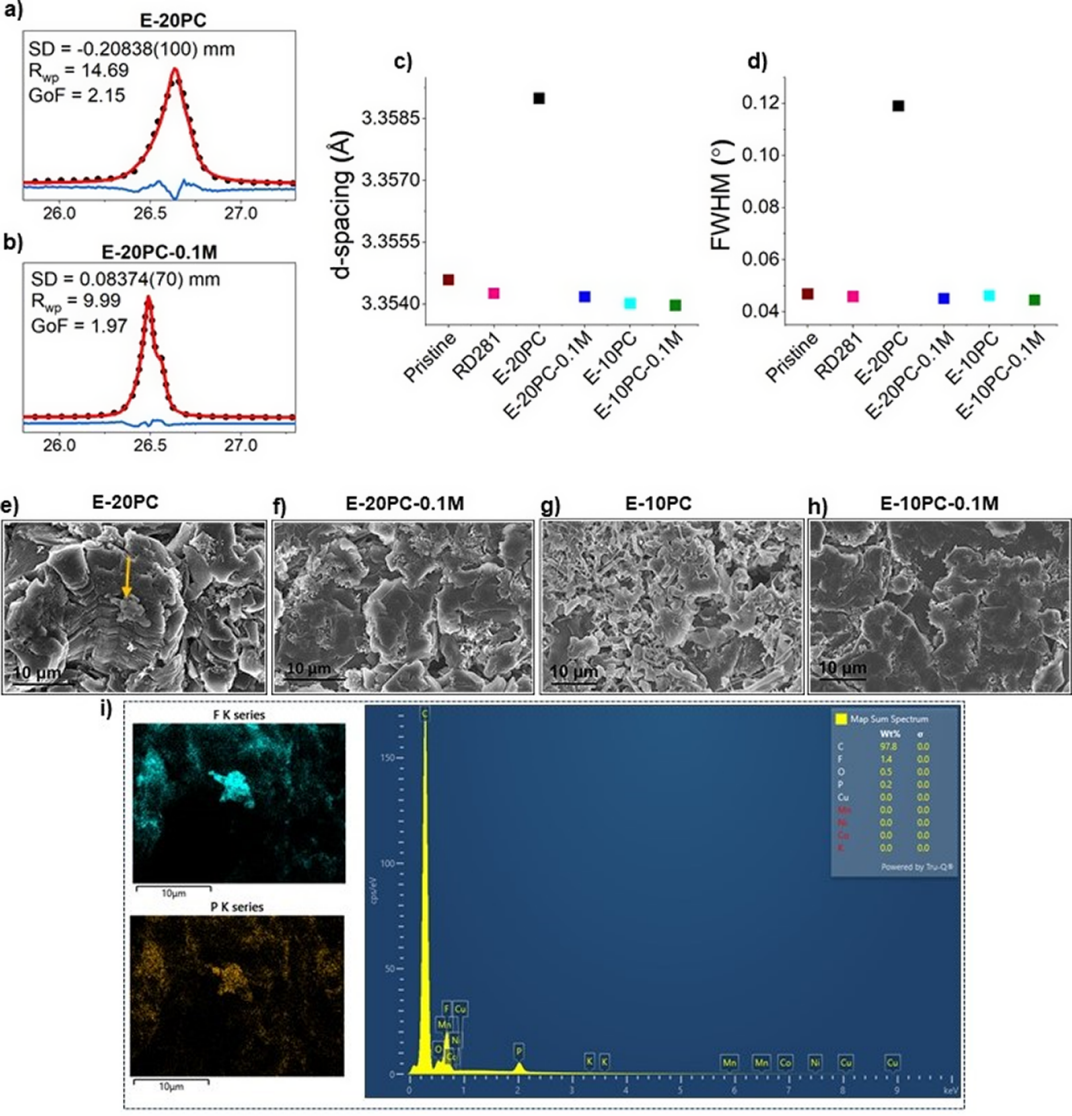
Suppression of exfoliation and Li dendrite on graphite anode in NMC 622|graphite cell. XRD diffraction refinement plot showing sample displacement (SD), *R*_wp_ (profile weighted residual value), GoF (goodness of fit) of graphite anode cycled with (a) E-20PC and (b) E-20PC-0.1M. Comparison of (c) *d*-spacing and (d) fwhm of graphite anode cycled with different electrolytes. SEM images of anode in (e) E-20PC, (f) E-20PC-0.1M, (g) E-10PC, (h) E-10PC-0.1 M after 100 cycles, showing graphite exfoliation (in E-20PC), Li deposition (in E-10PC) and their mitigation (in E-20PC-0.1 and E-10PC-0.1 M, respectively), and (i) EDX mapping and spectra of anode cycled with E-20PC electrolyte confirming electrolyte decomposition products, i.e., F and P.

A probable mechanism of extensive electrolyte decomposition (Figure 1g) in E-20PC and E-10PC is graphite exfoliation, which supplies continuous fresh active materials to the electrolyte. Therefore, X-ray diffraction (XRD) was conducted on cycled graphite anodes exhibiting superior and inferior performances. The interlayer spacing (*d*-spacing) of graphite is obtained from the 002 reflection and compared to the anode cycled with commercial electrolyte (RD281) along with a pristine anode for reference. The 002 reflection of the pristine graphite anode presented in Figure S5 results in an interlayer *d*-spacing of 3.354 Å in Figure 3c, which matches with the reported values in the literature.^33,34^ The refinement of graphite cycled with RD281 yields *d*-spacing of 3.354 Å and fwhm of 0.046° close to the value obtained from pristine graphite, indicating the intact graphite layers with no exfoliation upon cycling with the commercial electrolyte. However, a relatively broad 002 reflection is seen for E-20PC (Figure 3a), with a higher *d*-spacing of 3.359 Å (Figure 3c) and fwhm of 0.12° (Figure 3d). This confirms the exfoliation of graphite layers in the E-20PC electrolyte.^35^ Moving on to the E-20PC-0.1 M electrolyte, the obtained *d*-spacing of 3.354 Å (Figure 3c) and fwhm of 0.045° (Figure 3d) confirm the intactness of the graphite layers. Nevertheless, with the addition of 0.1 M KPF_6,_ a significant decrease in *d*-spacing and fwhm is observed, demonstrating that the exfoliation of graphite is suppressed. Further corroboration is seen in the SEM image in Figure 3e that shows the exfoliated graphite layers in E-20PC, which is prohibited by the introduction of 0.1 M KPF_6_ (E-20PC-0.1M) in Figure 3f. Figure S6 shows the SEM images of graphite exfoliation with regard to different concentrations of KPF_6_ additive in 20 vol % PC electrolytes. Electrolyte decomposition products are also identified on the exfoliated layers, presented as the orange arrow in Figure 3e. The EDX mapping of the corresponding SEM image is presented in Figure 3i, revealing the presence of electrolyte decomposition products, i.e., F and P. This ensures the deposition of electrolyte decomposition products on the edges of the exfoliated graphite.

In the case of E-10PC (Figure S5), the refined *d*-spacing and fwhm are in close proximity to those of pristine graphite, demonstrating no sign of exfoliation in graphite layers. However, Li^0^ (metallic Li) deposition observed in Figure 2e is the sole and predominant cause for electrolyte decomposition in E-10PC, which is witnessed as a dendrite cluster on the graphite surface in Figure 3g. The incorporation of 0.1 M KPF_6_ additive (E-10PC-0.1 M) mitigates the dendritic growth as seen in Figures 2e and 3h, delivering higher discharge capacity and CE. Figure S7 denotes the impact of KPF_6_ additive concentrations on Li dendrite formation in 10 vol % PC electrolytes.

### 3.3 Effect of Increasing KPF_6_ Concentration

**Figure 4:**
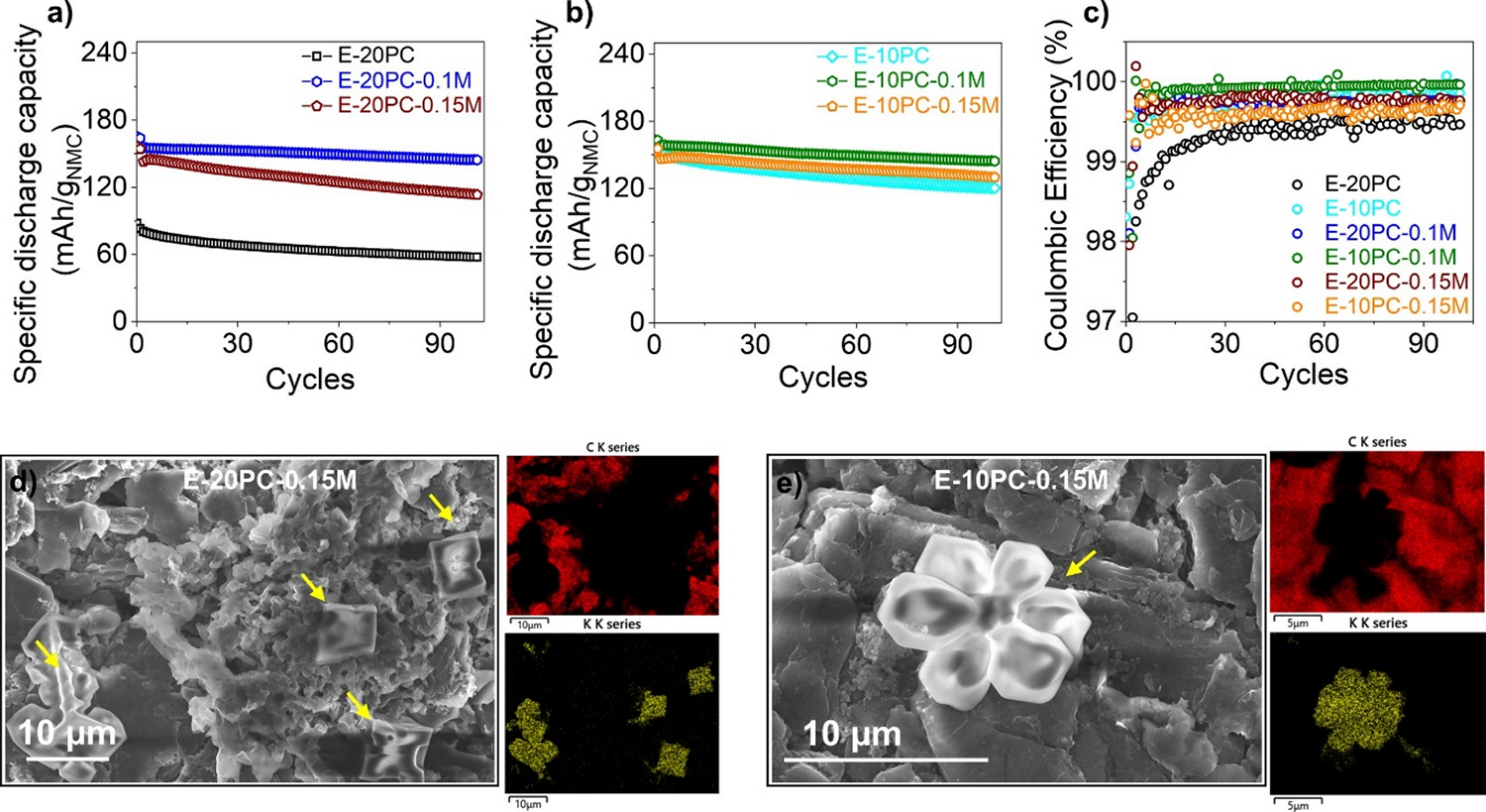
Effect of increasing the additive content to 0.15 M in NMC 622 | graphite. Cycling stability of (a) 20 vol % PC, (b) 10 vol % PC, and (c) CE comparison of NMC 622|graphite in different electrolytes. SEM imaging and corresponding EDX elemental mapping of carbon and potassium of graphite anode in (d) E-20PC-0.15 M and (e) E-10PC-0.15 M electrolyte after 100 cycles, presented potassium dendrite formation.

0.1 M KPF_6_ (0.1 M) is found to be the optimum concentration in 20 and 10 vol % PC electrolytes, which inhibits graphite exfoliation and Li dendrite formation, respectively. However, the electrochemical performance suffers as the concentration of KPF_6_ is increased to 0.15 M. The specific discharge capacities and CE decline in both 20 and 10 vol % PC electrolytes as shown in Figure 4a–c (and d*Q*/d*V* vs *V* in Figure S8). Morphological investigation in SEM images and the corresponding EDX spectral mapping in Figure 4d,e denotes the presence of potassium dendrites on the graphite surface. The absence of carbon intensity on the deposited area ensures that the dendritic deposits cover the graphite surface fully while blocking the X-ray emanating from it. Potassium dendrite deposition diminishes the active surface area of graphite, hindering the Li ^+^ transportation. In addition, these also act as defect sites for further Li deposition and subsequent electrolyte decomposition, deteriorating the cell’s performance.

The morphological investigations of E-20PC-0.1 and E-10PC-0.1 M show no visible indication of potassium deposition on the graphite surface. However, for confirmation, the potassium deposition potential is calculated using the Nernst Equation with respect to Li/Li ^+^ as follows.
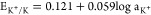
1

Eq 1 shows the potassium deposition potential at room temperature.^28,36^ The calculated potassium deposition potential with respect to the additive concentration is presented in Table S2. This means that potassium deposition occurs when the graphite anode reaches this potential. Figure S9 confirms the maximum potential achieved by graphite is ∼0.08 V in 0.1 M KPF_6_ concentration, which is higher than the K deposition potential. This ensures that potassium dendrites are absent in 0.1 M KPF_6_ concentration, i.e., in E-20PC-0.1 and E-10PC-0.1 M electrolytes. Therefore, potassium remains in the K ^+^ form, and its solvation shell directs the SEI formation via enhanced diffusion through E-20PC-0.1 and E-10PC-0.1 M electrolytes. Additionally, the lower desolvation energy of K ^+^ solvation shell^37,38^ produces the very initial SEI film, diminishing PC decomposition and cointercalation, which is elaborately discussed in the later section.

### 3.4 SEI Chemistry Characterization

**Figure 5:**
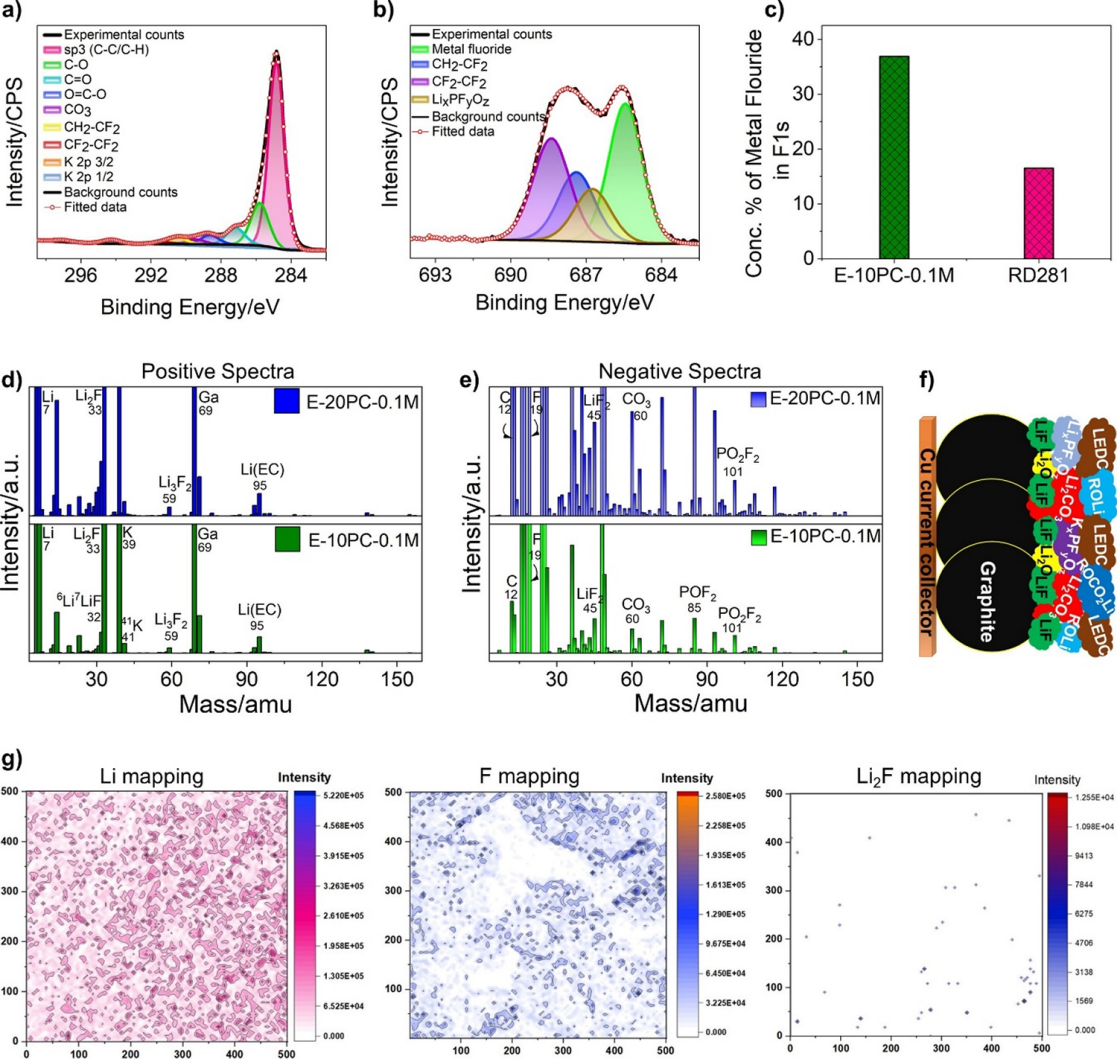
Characterization of SEI components produced on graphite anode with optimum electrolyte. XPS spectra of (a) C 1s and (b) F 1s of graphite with E-10PC-0.1 M. (c) Concentration percentage comparison of metal fluoride in the F 1s spectrum on graphite with E-10PC-0.1 M and RD281. SIMS (d) positive ion and (e) negative ion mass spectra comparison of graphite surface after cycling with E-20PC-0.1 and E-10PC-0.1 M. (f) Schematic illustration of SEI components on graphite cycled with E-10PC-0.1 M. (g) SIMS maps comparison of ^7^Li ^+^, ^19^F ^–^, and ^7^Li_2_F ^+^ ion fragments on graphite surface after cycling with E-10PC-0.1 M.

Further characterizations are employed to investigate the interphase chemistry of SEI formed on the graphite anode with modified electrolytes in order to understand the mechanism behind enhanced cell performance. Figure 5a represents a deconvoluted C 1s spectrum consisting of a sharp sp^3^ C – C/C–H peak (284.8 eV) and electrolyte solvent decomposition products, i.e., C–O (∼285.7 eV), C=O (∼287.2 eV), O=C–O (∼288.7 eV), CO_3_ (∼289.5 eV) from EC [(CH_2_O)_2_CO ] and EMC [C_2_H_5_OCOOCH_3_ ] .^32^ The CH_2_ – CF_2_ (∼290.4 eV) and CF_2_ – CF_2_ (∼291.2 eV) peaks are from PVDF binder in the graphite coating and its further fluorination, respectively,^32^ which are also identified at ∼687.3 and ∼688.3 eV binding energy in the F 1s spectrum in Figure 5b. Two broad peaks at ∼294.2 and ∼297.0 eV toward the end of C 1s spectrum (Figure 5a) are assigned to the K 2p_3/2_ and K 2p_1/2_, respectively, which is confirmed by K 2s peak at ∼378 eV in Figure S10. The F 1s spectrum in Figure 5b exhibits two additional peaks at ∼685.4 and ∼686.8 eV attributed to metal fluoride and Li_*x*_PF_*y*_O_*z*_ (due to LiPF_6_ salt decomposition products on the graphite surface).^39^Figure 5c outlines the concentration percentage of metal fluoride (36.9 % ), which is substantially higher (almost double) in the optimized electrolyte; E-10PC-0.1 M compared to commercial electrolyte (16.5 %). It should be noted that the peak in the F 1s region at ∼685.4 eV is assigned to metal fluoride because of the likely presence of lithium fluoride (LiF) in addition to potassium fluoride (KF), arising from the LiPF_6_ primary and KPF_6_ additive salts, respectively.

To gain better insight into the composition of metal fluoride, secondary ion mass spectroscopy (SIMS) was carried out, as shown in Figure 5d,e. Each peak in Figure 5d,e corresponds to the ion cluster of compounds (organic and inorganic) present in the SEI layer on the graphite surface. Table S3 shows the list of the ion clusters collected on the graphite anode cycled with PC-based ternary electrolytes. ^7^Li ^+^ and ^19^F ^–^ are the dominant peaks in positive and negative ion spectra, as clearly evident by the corresponding SIMS maps in Figure 5g (compared to Li_2_F ^+^ in Figure 5g, and ^39^K ^+^, ^41^K ^+^ in Figure S11). The ion fragments associated with LiF are ^6^Li^7^Li ^+^ (32 amu), ^7^Li_2_F ^+^ (33 amu), ^7^Li_3_F ^+^ (40 amu), ^7^Li_2_F_2_^+^ (52 amu), ^6^Li^7^Li_2_F_2_^+^ (58 amu), and ^7^Li_3_F_2_^+^ (59 amu) observed in positive ion spectra in Figure 5d. Similarly, ^6^LiF_2_^–^ (44 amu), ^7^LiF_2_^–^ (45 amu), ^7^Li_2_F_3_^–^ (71 amu) negative ion fragments in Figure 5e confirm the existence of LiF in the SEI layer.^28,40−45^ In addition, K isotopes at 39 amu (^39^K ^+^ ) and 41 amu (^41^K ^+^ ) are also present in positive ion mode (Figure 5d). In order for KF to be present in the SEI, the corresponding ion fragments must be present in SIMS mass spectra. However, the ion species corresponding to KF, e.g., ^39^K_2_F ^+^ (97 amu), ^39^K^41^KF ^+^ (99 amu), ^39^K_3_F_2_^+^ (155 amu), and ^39^K_2_^41^KF_2_^+^ (157 amu) in positive ion and ^39^KF_2_^–^ (77 amu), ^41^KF_2_^–^ (79 amu), ^39^K_2_F_3_^–^ (135 amu), and ^39^K^41^KF_3_^–^ (137 amu) in negative ion spectra^28,37,46,47^ are not present, indicating the absence of KF. Although two small peaks are 97 and 99 amu in positive ion spectra, the intensity ratio (^99^*I*/^97^*I*) does not match with the relative abundance ratio of potassium isotopes (^39^K/^41^K = 13.8).^48^ SIMS was also repeated at the same location for the number of cycles; however, the intensity ratio of ^39^K/^41^K does not match with the intensity ratio at 97 and 99 amu, confirming these peaks are not ^39^K_2_F ^+^ and ^39^K^41^KF ^+^, respectively (Figure S12). This indicates that KF is absent as an SEI constituent. Therefore, the ion species at 97 and 99 amu could be C_5_H_5_O_2_^+^ and C_5_H_7_O_2_^+^_,_ respectively. This validates that the increase in concentration percentage of metal fluoride is due to higher LiF content in the optimized electrolyte; E-10PC-0.1 M.

LiF produces a compact and robust SEI film due to its lower solubility,^38,49,50^ inhibiting PC cointercalation and successive graphite exfoliation caused by this. The suppression of PC decomposition is the dominant reason for improving the electrochemical performance, which is primarily associated with the following factors.

0.1 M KPF_6_ additive enhances the fluoride (LiF) content in the SEI layer, investigated by XPS and SIMS spectra in Figure 5c,d, respectively. The robust LiF-rich SEI layer on the graphite surface due to the lower solubility of LiF^51^ ensures the stability of the film in PC-based electrolyte upon cycling. This stable SEI prevents the cointercalation of PC solvent into the graphite layers and, therefore, suppresses the subsequent graphite exfoliation. In addition, the insulating nature of LiF^51,52^ blocks the potential electron leakage from the defect sites,^28^ which inhibits any possible electrolyte decomposition as well as Li dendrite formation/growth—facilitating faster Li ^+^ transportation through graphite anode.^53,54^ Furthermore, LiF, an inorganic SEI compound is highly probable to be seen toward the graphite anode in the SEI layer (away from the electrolyte). Hence, increased LiF content acts as a perfect SEI constituent in obstructing the PC intercalation in order to overcome the exfoliation of the graphite anode.
It has been previously studied that the solvation shell of cations having lower Stokes radius compared to Li ^+^ (such as Cs ^+^ ) exhibits a faster diffusion rate and lower desolvation energy in the electrolyte compared to Li ^+^ solvation shell,^37,38^ facilitating the rapid formation of initial SEI layer at the electrode/electrolyte interphase.^24^ Herein, K ^+^ solvation shell (due to lower Stokes radius compared to Li ^+^ ) produces the very initial SEI layer with higher LiF content in PC-based electrolyte. As previously mentioned, the intrinsic properties of LiF restrict PC decomposition and its cointercalation into the graphite anode, hence suppressing the graphite exfoliation and Li dendrite formation, thereby improving the electrochemical performance of the cell.

A schematic illustration is shown in Figure 5f, demonstrating higher LiF content in SEI layer, which improves cyclability of graphite anode in optimized PC-based ternary electrolyte, i.e., E-10PC-0.1 M.

## 4 Conclusions

In summary, a ternary electrolyte is formulated with PC, EC, and EMC solvents along with LiPF_6_ as the main salt and KPF_6_ as an additive to acquire optimized properties of the solvents. The concentration of KPF_6_ electrolyte additive with volume percentage of PC solvent is systematically investigated and analyzed alongside a commercial electrolyte. It is found that 0.1 M KPF_6_ suppresses not only the exfoliation of graphite layers but also Li dendrite formation, maintaining the cyclability in PC-based electrolytes. The faster diffusion rate and lower desolvation energy of the K ^+^ solvation shell produce the very initial LiF-rich SEI film, decreasing PC decomposition on the graphite surface, thereby minimizing the probability of graphite exfoliation. In addition, the strong, insoluble SEI layer with higher LiF content maintains the structural integrity of graphite anode in optimized PC-based electrolyte; i.e., E-10PC-0.1 M. Furthermore, the electron blocking capacity of LiF-rich SEI layer eliminates the risk for Li dendrite formation, resulting in enhanced electrochemical performance.

## Supporting Information Available

The Supporting Information contains experimental procedures, supplementary Figures S1–S12 showing d*Q*/d*V* vs *V* plots, reproducibility of electrochemical performance, comparison of specific capacities and CEs of all the electrolytes, XRD refinements, electrochemical performance, and SEM images when concentration increased to 0.15 M, anode potential detection by three-electrode cell, XPS spectra, SIMS maps, and spectra for number of cycles, and supplementary Tables S1–S4 providing electrolytes used for the study, potassium deposition potential on graphite with respect to additive, SIMS ion clusters collected from graphite, and the properties of the electrodes (PDF)
