# Supplementary material for: Combined Stabilizing of the Solid–Electrolyte Interphase with Suppression of Graphite Exfoliation via Additive-Solvent Optimization in Li-Ion Batteries
Source: ACS Appl Mater Interfaces. 2023 Oct 18;15(43):50185–95. doi: 10.1021/acsami.3c10792 (PMC10623506; doi:10.1021/acsami.3c10792)
Supplement: Supplementary file 1 — am3c10792_si_001.pdf [file am3c10792_si_001.pdf]

Supporting Information

Combined stabilising of the solid-electrolyte interphase with suppression of graphite exfoliation via additive-solvent optimisation in Li-ion batteries.

Sanghamitra Moharana*[1], Geoff West[1], Ashok S. Menon[1], Wilgner Lima da Silva[1,2], Marc Walker[3], Melanie J. Loveridge[1]

[1] Warwick Manufacturing Group (WMG), University of Warwick, Coventry, CV4 7AL, UK.

[2] Department of Chemistry, University of Warwick, Coventry, CV4 7AL, UK.

[3] Department of Physics, University of Warwick, Coventry, CV4 7AL, UK.

Corresponding Author Email: k.moharana@warwick.ac.uk

Table S1. The list of formulated electrolytes used for this study.

**Electrolyte Nomenclature**	**Electrolyte composition**
E-20PC	EC: PC: EMC (1:2:7 v/v/v), 1M $LiPF_6$, 1 wt% VC
E-20PC-0.001M	EC: PC: EMC (1:2:7 v/v/v), 1M $LiPF_6$, 1 wt% VC, 0.001M KPF_6
E-20PC-0.01M	EC: PC: EMC (1:2:7 v/v/v), 1M $LiPF_6$, 1 wt% VC, 0.01M KPF_6
E-20PC-0.1M	EC: PC: EMC (1:2:7 v/v/v), 1M $LiPF_6$, 1 wt% VC, 0.1M KPF_6
E-20PC-0.15M	EC: PC: EMC (1:2:7 v/v/v), 1M $LiPF_6$, 1 wt% VC, 0.15M KPF_6
E-10PC	EC: PC: EMC (2:1:7 v/v/v), 1M $LiPF_6$, 1 wt% VC
E-10PC-0.001M	EC: PC: EMC (2:1:7 v/v/v), 1M $LiPF_6$, 1 wt% VC, 0.001M KPF_6
E-10PC-0.01M	EC: PC: EMC (2:1:7 v/v/v), 1M $LiPF_6$, 1 wt% VC, 0.01M KPF_6
E-10PC-0.1M	EC: PC: EMC (2:1:7 v/v/v), 1M $LiPF_6$, 1 wt% VC, 0.1M KPF_6
E-10PC-0.15M	EC: PC: EMC (2:1:7 v/v/v), 1M $LiPF_6$, 1 wt% VC, 0.15M KPF_6
RD281	EC: EMC (3:7 v/v), 1M $LiPF_6$, 1 wt% VC (commercial electrolyte)

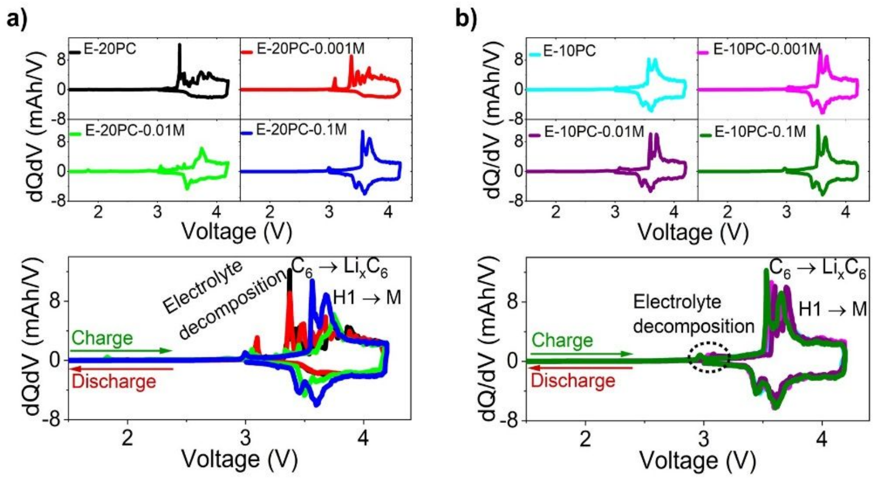

Figure S1. dQ/dV vs V plot of graphite | NMC 622 full cell with various concentrations of KPF_6 additive in (a) 20PC and (b) 10PC based electrolytes.

Incremental capacity plot for E-20PC (without KPF_6 additive) and E-20PC-0.001M show crowded peaks during charging with no reduction peak during discharging in Figure S1a. This signifies that Li^+ ions are consumed through electrolyte decomposition, delivering poor specific capacities in Figure 1a. Moreover, well defined two oxidation and two corresponding reduction peaks are monitored with the increase in additive content to 0.1M, which perfectly aligns with the dQ/dV vs V peak of commercial RD281 electrolyte [1], confirming Li^+ lithiation and delithiation through graphite layers has occurred in E-20PC-0.1M electrolyte.

In Figure S1b, E-10PC shows two sharp oxidation and reduction dQ/dV vs V peaks without the additive, demonstrating Li^+ intercalation and de-intercalation through the graphite anode, unlike the E-20PC electrolyte. It is observed that the oxidation peak attributed to Li^+ intercalation becomes sharper and eventually shifts towards the left with increasing in additive content to 0.1M in 10PC electrolytes, ensuring reduced polarisation compared to other electrolytes.

Moreover, Figure S1 denotes that the addition of 0.1M KPF_6 reduces electrolyte decomposition and polarisation in 20PC and 10PC respectively, improving cells performance upon cycling.

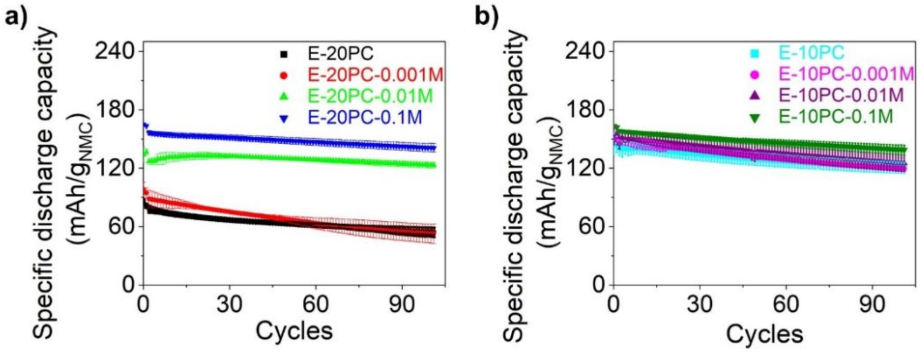

Figure S2. Reproducible cyclic stability of graphite | NMC 622 full cell using various concentrations of KPF_6 in (a) 20PC, (b) 10PC electrolytes.

For data reproducibility, three cells of the same electrodes and electrolytes have been tested under the same experimental condition. It is observed that the long-term cycling data of graphite | NMC 622 full cell using additive-based electrolytes in Figures S2a and b are highly reproducible, irrespective of additive concentrations. This ensures the stable performance of cells with modified electrolytes.

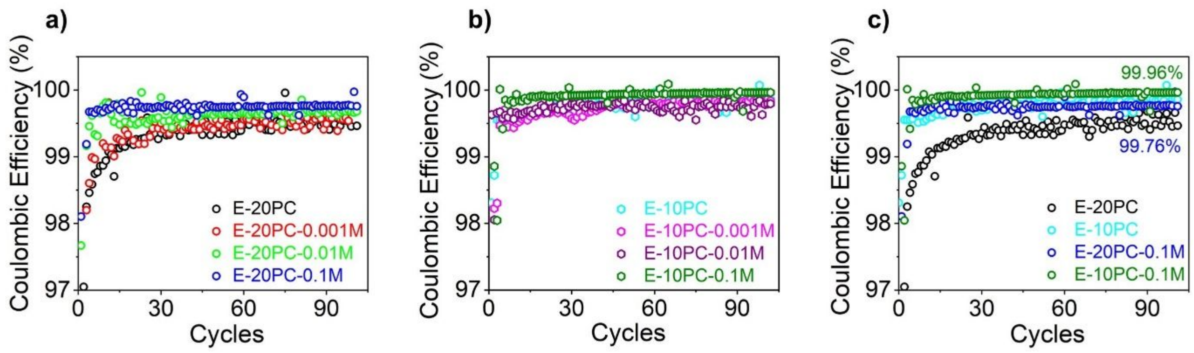

Figure S3. Coulombic Efficiencies (CEs) of graphite | NMC 622 full cell cycled with various concentrations of KPF_6 in (a) 20PC, (b) 10PC electrolytes. (c) CEs comparison of optimal E-10PC-0.1M, E-20PC-0.1M electrolytes with E-20PC and E-10PC (without KPF_6) electrolytes.

The graphite | NMC 622 full cells with an optimal concentration of 0.1M KPF_6 show higher CEs compared to other modified electrolytes. The enhanced CE is maintained up to 100 cycles with 0.1M KPF_6 concentration irrespective of PC content, shown in Figure S3a and b. The cyclability is retained thereby delivering the highest CE of 99.96% in E-10PC-0.1M electrolyte in comparison with E-20PC-0.1M (CE of 99.76%), E-10PC (CE of 99.86%) and E-20PC (CE of 99.46%) shown in Figure S3c.

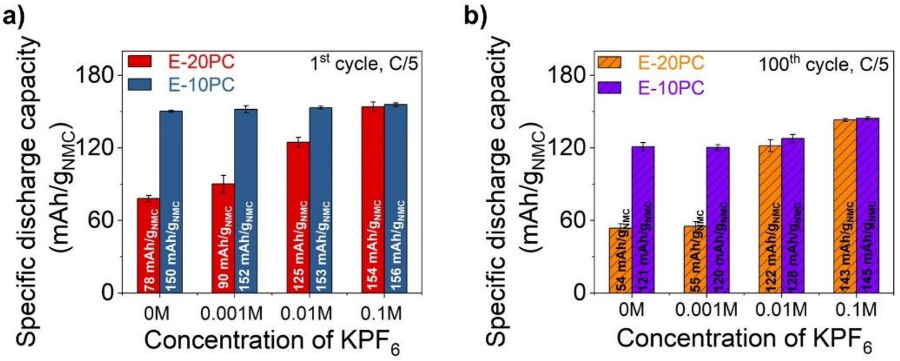

Figure S4. Comparison of specific discharge capacities of NMC 622 | graphite full cell with respect to the concentration of KPF_6 additive for 20PC and 10PC electrolytes.

For a clearer understanding, a comparison of specific discharge capacities with respect to the concentration of KPF_6 additive for 20PC and 10PC are presented in Figure S4a and b. It is observed that the specific discharge capacity is doubled when the PC content is decreased from 20 vol% to 10 vol% in the very first cycle (Figure S4a) and maintained the similar capacity difference even on 100th cycle (Figure S4b). This demonstrates that the specific discharge capacities are majorly affected by the PC content in the electrolyte. High PC content i.e., 20 vol% produces more soluble decomposition products (lithium propylene dicarbonate, LPDC) [2], hence, builds a loose SEI film on graphite anode. Therefore, the probability of graphite exfoliation via PC co-intercalation is high in 20PC electrolyte, leading to the poor electrochemical performance [3–5] compared to 10PC based electrolytes.

With introduction of KPF_6 additive into the electrolyte, an increase in specific discharge capacities is obtained in both 20PC and 10PC electrolytes. In addition to this, the difference in specific discharge capacities (between cells cycled with 20PC and 10PC electrolytes) is decreased, when KPF_6 concentration is increased to 0.01M. Lastly, the difference in capacities is reduced to ~ 1 mAh/g_{NMC} when 0.1M KPF_6 is added to the 20PC and 10PC electrolytes. The

similar trend is observed on the 100th cycle, presented in Figure S4b, indicating the stable cyclability of the cell using modified electrolytes.

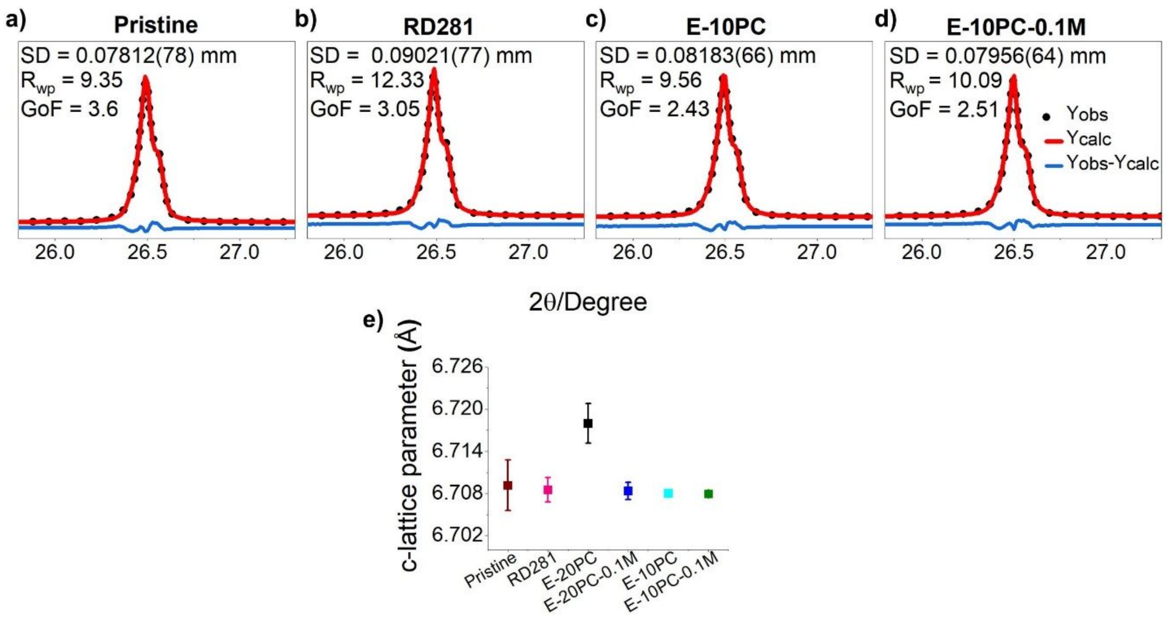

Figure S5. XRD diffraction refinement plots of (a) Pristine graphite, and graphite anodes cycled with (b) RD281, (c) E-10PC, (d) E-10PC-0.1M electrolytes for 100 cycles. (e) Comparison of c-lattice parameter of graphite after refinement.

XRD diffraction refinement plots of graphite anode cycled with RD281, E-10PC, E-10PC-0.1M electrolytes are presented Figure S5. For a comparison, the refinement of pristine graphite anode is carried out in Figure S5a. The refinement provides the c-lattice spacing of graphite, from which interlayer d-spacing is obatinaed. The refinement results in c-lattice parameter of 6.71798(280) Å for E-20PC in electrolyte Figure S5e, suggesting the expansion of graphite layers [6]. However, the c-lattice parameter for E-10PC, E-20PC-0.1M and E-10PC-0.1M are 6.70803(8) Å, 6.70836(120) Å and 6.707947(85) Å respectively, which is close to the value obtained from the pristine graphite i.e., 6.70917(358) Å. This confirms the presence of relatively stable SEI film in E-20PC-0.1M and E-10PC-0.1M electrolytes, which suppresses in PC co-intercalation via subsequent graphite exfoliation (due to 0.1M KPF_6 electrolyte additive).

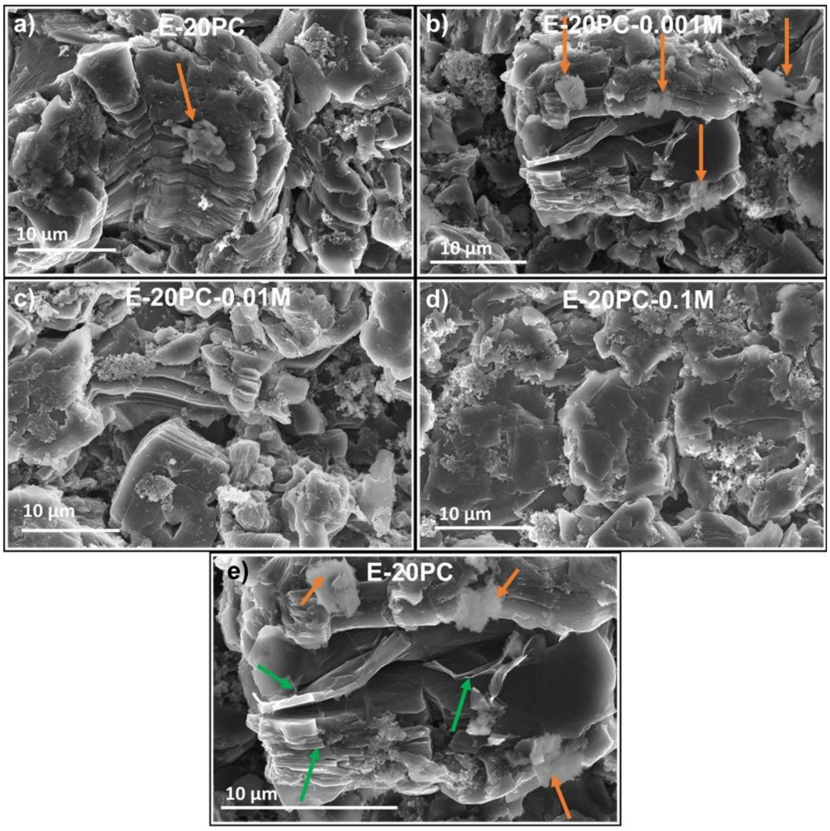

Figure S6. SEM images of graphite anodes cycled in 20PC electrolytes containing (a) 0M, (b) 0.001M, (c) 0.01M, and (d) 0.1M KPF_6 additive. (e) High magnification (5000x) SEM image presenting graphite edges (green arrow) and mossy like deposits (orange arrow).

The impact of KPF_6 additive concentrations on graphite anode in 20PC electrolytes is presented in Figure S6. The exfoliated graphite layers are clearly evident in Figure S6a and e. PC solvated Li^+ ions co-intercalates into the graphite layers and further decomposes to gaseoue products, which acts as a source of graphite exfoliation [7]. The exfoliation appears to be reduced with the incorporation of KPF_6 additive in Figure S6b and c. Ultimately, with 0.1M concentration, graphite exfoliation is fully suppressed, which aligns with the XRD results in Figure 3c and d.

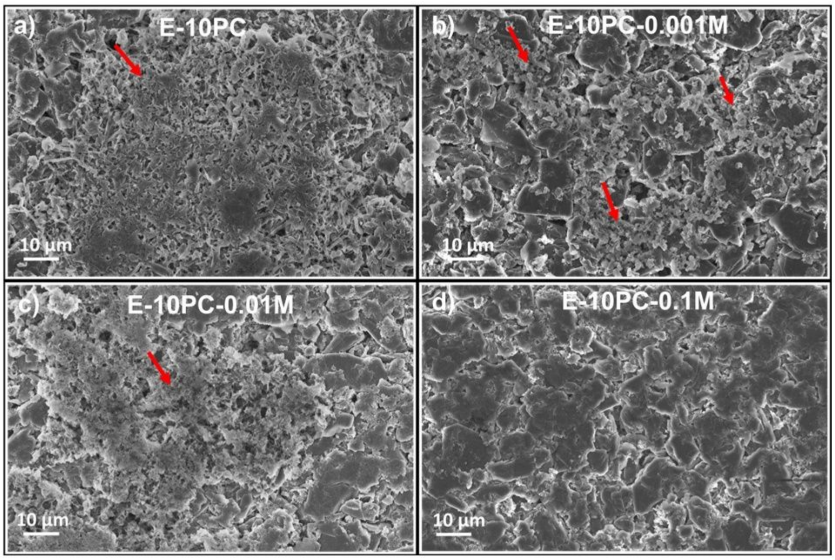

Figure S7. SEM images of graphite anodes (pressenting Li metal deposition in red arrow) cycled in 10PC electrolytes containing (a) 0M, (b) 0.001M, (c) 0.01M, and (d) 0.1M KPF_6 additive.

The impact of KPF_6 additive concentrations on graphite anode in 10PC electrolyte is presented in Figure S7. A large cluster of Li dendrites is observed (presented as red arrow) on graphite surface in E-10PC (Figure S7a). This deposition of Li dendrite is also detected electrochemically in Figure 2e, when graphite potential falls below 0 V vs Li/Li^+. Li deposition is seemingly reduced, when the additive concentration increases from 0.001M to 0.01M (Figure S7b and c). Lastly, 0.1M KPF_6 in E-10PC-0.1M electrolyte, Li dendrites appears to be prevented in Figure S7d, which is consistent with the electrochemical cycling result presented in Figure 2e.

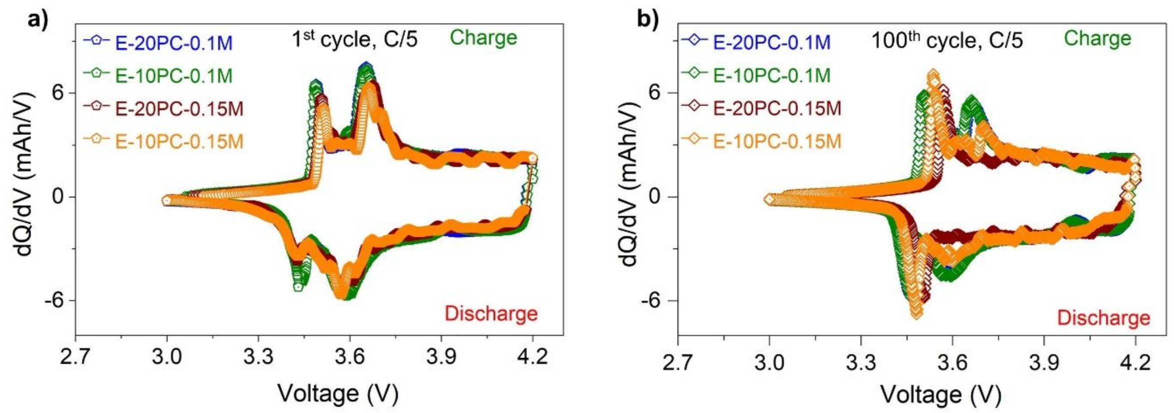

Figure S8. Incremental capacity plot comparison of (a) 1st cycle, and (b) 100th cycle of NMC 622 | graphite full cell cycled with E-20PC-0.1M, E-10PC-0.1M, E-20PC-0.15M, E-10PC-0.15M electrolytes.

A comparison of incremental capacity plots for 1st cycle and 100th cycle are presented in Figure S8. The dQ/dV vs V peaks for optimum concentrations E-20PC-0.1M and E-10PC-0.1M (in both 1st and 100th cycles) completely overlaps on each other, indicating no significant resistance building up in both the electrolytes. Two oxidation peaks i.e., (a) graphite lithiation and (b) NMC phase transition and its corresponding two eductions peaks are detected as shown in Figure 1b, e and Figure S1.

Moving on to 0.15M KPF_6, four dQ/dV vs V peaks are observed identical to E-20PC-0.1M and E-10PC-0.1M during 1st cycle in Figure S8a. However, dQ/dV vs V plot for 100th cycle in Figure S8b is found to have single peak both in charging and discharging processes especially in E-20PC-0.15M. Similar behaviour have been reported previously [1] with KPF_6 modified electrolytes. Metallic potassium deposition is believed to be the cause of this behaviour.

Table S2. Potassium deposition potential with respect to the concentration of KPF_6 additive in the electrolytes.

Concentration of KPF_6 additive (M)	Potassium deposition potential on graphite anode (V)
0.001M	-0.056
0.01M	0.003
0.1M	0.062
0.15M	0.072

Table S2 shows the equillibrium potassium deposition potential with respect to the concentrations of KPF_6 additive in the electrolytes. This denotes that potassium metal deposition starts when graphite anode achieves these potentials.

The potassium deposition potential for optimum 0.1M KPF_6 concentration is 0.062 V. However, graphite potentials achieved by 0.1M KPF_6 additive (in E-20PC-0.1M and E-10PC-0.1M) are 0.07 V and 0.08 V (See, Figure S9b), ensuring no metallic potassium deposition on graphite anode. This confirms that potassium in ionic form K^+ assists in building stable SEI in 20PC and 10PC based electrolytes.

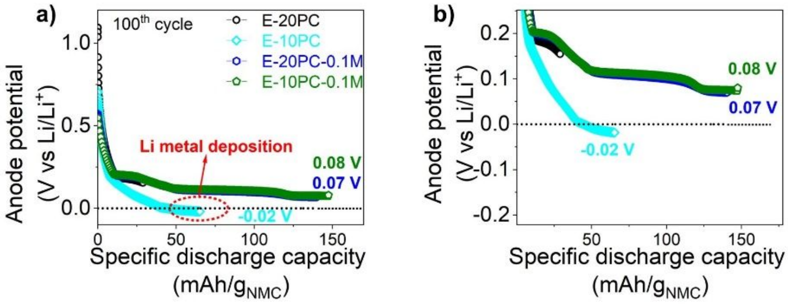

Figure S9. (a) Three electrode EL-cell graphite potential (graphite | Li) comparison of without additive (E-20PC, and E-10PC) and with KPF_6 additive based electrolytes (E-20PC-0.1M and E-10PC-0.1M) at C/5 cycling during 100th cycle. (b) zoomed-in cycling data.

Three electrode El-cells were assembled for monitoring graphite anode potnetial upon cycling. Figure S9a shows the comparison of graphite potentials using different electrolytes presenting inferior (E-20PC and E-10PC) and superior (E-20PC-0.1M and E-10PC-0.1M) performances. A zoomed-in plot is presented in Figure S9a. The specific discharge capacity of E-20PC is significantly lower compared to other electrolytes cause by excessive electrolyte decomposition via graphite exfoliation, as shown in Figure 1a, 2a and, 3c. In E-10PC, a negative graphite potential (-0.02V) is measured, indicating the deposition of Li metal due to the polarisation developed in the cell. However, a significantly high specific discharge capacity of ~ 150 mAh/g_{NMC} is observed when 0.1M KPF_6 is added into 20PC and also 10PC electrolytes. E-20PC-0.1M achieves a positive graphite potential of ~ 0.07 V by suppressing graphite exfoliation (see, Figure 3c and d). Similarly, E-10PC-0.1M achieves a positive potential of ~ 0.08 V by inhibiting Li metal deposition (also shown in Figure 3h). In addition, graphite potential for E-20PC-0.1M and E-10PC-0.1M electrolytes is higher than the potassium deposition potential (0.062 V for 0.1M KPF_6), indicating no sign of potassium deposition on graphite anode. This reveals the enhanced diffusion of the K^+ solvation shell (potassium in ion form), due to the smaller stokes radius (compared to Li^+) [8,9] assists in constructing stable SEI

to inhibit graphite exfoliation and polarisation. Therefore, 0.1M KPF_6 is proved as an optimum additive concentration for PC based electrolytes.

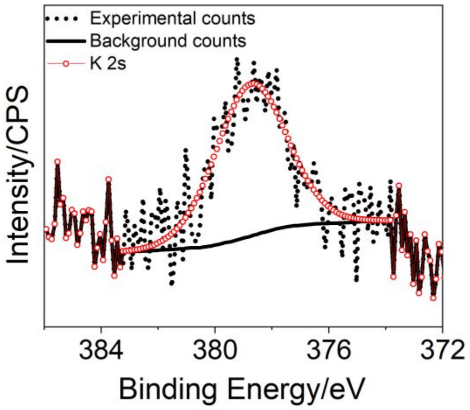

Figure S10. XPS K 2s spectra of graphite anode cycled with E-10PC-0.1M – confirming the presence of potassium element on the anode surface.

K 2*s* peak is detected at ~ 378 eV binding energy [10] as shown in Figure S10. This confirms the tiny broad peaks as K $2p_{3/2}$ (~ 294.2 eV) and K $2p_{1/2}$ (~ 297.0 eV) respectively, towards the end of the C 1s spectrum in Figure 5a.

Table S3. SIMS ion fragments collected from graphite anode cycled with E-20PC-0.1M, and E-10PC-0.1M electrolytes.

Mass (amu)	Positive Ion fragments	Mass (amu)	Negative ion fragments
6	^{6}Li	6	^{6}Li
7	^{7}Li	7	^{7}Li
12	C	12	C
13	CH	13	CH
14	N/Li_2	14	N/CH_2
15	CH_3, Li_2H	15	CH_3
16	O	16	O
17	OH	17	OH
19	F from $LiPF_6$/LiF	19	F
23	LiO	22	^{6}LiO
24	LiOH	23	LiO
26	C_2H_2	24	LiOH, C_2
27	C_2H_3	25	C_2H
28	Si	26	C_2H_2, CN
29	C_2H_5, COH	28	Si, CO
30	Li_2O	31	OCH_3, P, CF

31	Li_2OH, CF, C_2H_7	32	O_2, S
**32**	**$^6Li^7LiF$**, O_2	33	SH
**33**	**Li_2F**	35	OF
37	Li_3O	36	C_3
39	LiO_2, ^{39}K	37	C_3H, H_2OF
**40**	**Li_3F**	38	F_2
41	C_3H_5, H_3F_2 From PVDF, ^{41}K	39	F_2H
43	C_3H_7, C_3H3O From PVDF	40	C_2O, LiO_2H
45	C_2H_5O	41	C_2HO, Li $(OH)_2$
46	Li_3N, Li_2O_2	42	CNO, C_2H_2O
51	C_4H_3, CHF_2 From PVDF	43	$C_2H_3O_2$, C_2F
**52**	**Li_2F_2**	**44**	CO_2, **$^6LiF_2$**
53	C_2H_6OLi	**45**	**LiF_2**, CO_2H
55	C_4H_7	47	PO
57	C_4H_9	48	C_4, SO, $(LiOH)_2$
**58**	**$^6Li^7Li_2F_2$**	49	C_4H
**59**	**Li_3F_2**	50	CF_2
63	C_4H_8Li	54	LiPO
67	Li_5O_2	55	C_2P, LiO_3

69	Ga	56	LiO_3H
71	LiO_4	57	CF_3
74	Li_2CO_3	58	Li $(OH)_3$
77	$C_2H_5O_3$, $C_3H_3F_2$ From PVDF	59	$C_2H_3O_2$
81	Li_3CO_3	60	CO_3
**85**	**Li_4F_3**	61	HCO_3
89	EC+H	63	PO_2
93	Li (VC)	**64**	HPO_2, **LiF_3**, SO_2
95	Li (EC), $C_2H_2F_3$ from PVDF	65	$Li_2(OH)_3$
97	$C_5H_5O_2$	66	C_3NO
99	$C_5H_7O_2$	67	$LiCO_3$
102	Li_2(EC)	70	$LiPO_2$
103	EC+$CH_{3,}$ PC+H	**71**	**Li_2F_3**, LiO_4
105	EMC+H	72	C_6
109	Li_3(EC), Li (PC)	74	C_5N
111	Li (EMC)	79	PO_3
118	Li_2(EMC)	80	SO_3
125	Li_3(EMC)	81	SO_3H
127	$C_2H_2O_5Li_3$	82	PFO_2

129	EC+C_3H_5	85	POF_2
131	EC+C_2H_3O	87	EC-H/$C_3H_3O_3$
159	Li_2+PF_6	**88**	**Li_3F**
		89	$Li_3(OH)_4$
		95	PO_4
		96	SO_4
		97	H_2PO_4
		100	SO_4
		101	PO_2F_2, $C_3H_2O_2P$
		102	$LiPO_4$
		103	EMC-H
		105	EC+OH
		113	$Li_4(OH)_5$
		121	EMC+OH
		123	$C_2H_4PO_4$
		127	Li $(CO_3)_2$
		131	EC+C_2H_3O
		137	$Li_5(OH)_6$, $C_3H_6PO_4$
		145	PF_6

		147	EMC+C_2H_3O
		149	CF_3SO_3

Table S3 demonstrates the ion fragments collected from the surface of the graphite anode cycled with with E-20PC-0.1M, and E-10PC-0.1M electrolytes. The ion fragments corresponding to lithium fluoride (LiF)[1] are presented in blue colour and bold letters in above Table S3.

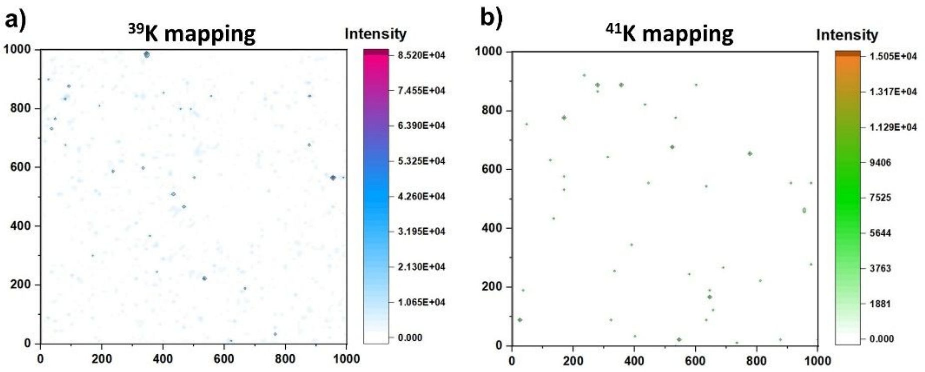

Figure S11. SIMS maps of secondary ion fragments of (a) $^{39}K^+$ and (b) $^{41}K^+$ on graphite surface cycled with E-10PC-0.1M electrolyte.

The SIMS maps of $^{39}K^+$ and $^{41}K^+$ ion species in positive ion modes are presented in Figure S11a and b. It is observed that $^{7}Li^+$ and $^{19}F^-$ secondary ion fragments (shown in Figure 5g) dominate the graphite surface in positive and negative ion modes through electrolyte decomposition, compared to $^{39}K^+$ and $^{41}K^+$ ion species.

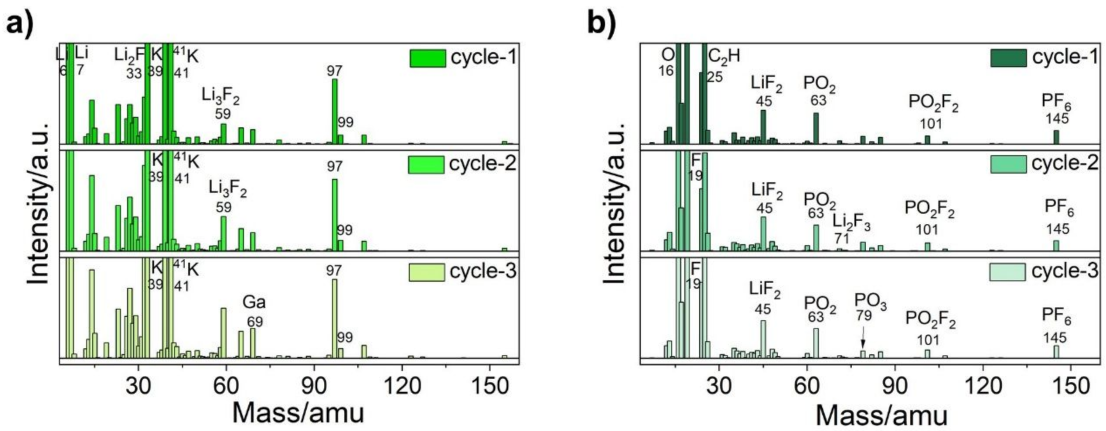

Figure S12. SIMS (a) positive ion and (b) negative ion mode repeated mass spectra for a number of cycles (at the same location) on cycled graphite surface cycled with E-10PC-0.1M electrolyte.

The mass spectra were repeated for several cycles at the same location on the graphite surface to collect ion species associated with potassium fluoride (KF). Figure S12a shows the positive ion mode mass spectra, where two peaks are detected at 97 and 99 amu. This could be attributed to $^{39}K_2F^+$ (97 amu) and $^{39}K^{41}KF^+$ (99 amu). Similarly, tiny peaks observed at 155 amu and 157 amu could be associated with $^{39}K_3F_2^+$ (155 amu) and $^{39}K_2{}^{41}KF_2^+$ (157 amu). However, the intensity ratios ($^{97}I/^{99}I$ and $^{155}I/^{157}I$) of $^{39}K_2F^+$ (97 amu)/ $^{39}K^{41}KF^+$ (99 amu) and $^{39}K_3F_2^+$ (155 amu)/ $^{39}K_2{}^{41}KF_2^+$ (157 amu) does not match with the relative abundance ratio of potassium isotopes ($^{39}K/^{41}K = 13.8$) [11], indicating the absence of KF in the SEI layer.

Similarly, on negative ion mode in Figure S12b, no peak is detected at 77 amu corresponding to $^{39}KF_2^-$ ion fragment, confirming the absence of KF in the SEI layer.

Table S4. Properties of MagE3 graphite anode and NMC 622 cathode.

	**Artificial Graphite**	**NMC 622**
Electrode Composition	91.83 wt% Hitachi MagE3<br>2 wt% Timcal C45 carbon<br>6 wt% Kureha 9300 PVDF Binder<br>0.17 wt% Oxalic Acid	90 wt% Targray NMC 622<br>5 wt% Timcal C-45<br>5 wt% Solvay 5130 PDVF Binder
Current Collector foil	Copper (Cu)	Aluminium (Al)
Foil Thickness (µm)	10	20
Total Electrode Thickness (µm)	52	58
Total Coating Thickness (µm)	42	38
Calendared Electrode Porosity	30.3%	37.1%
Total Coating loading (mg/cm^2)	6.35	9.78
Total Coating density (g/cm^3)	1.51	2.57
Reversible Capacity @~1C (RT) (mAh/cm^2)	1.92	1.41
Specific Reversible Capacity @~1C (RT) (mAh/g)	330	160

REFERENCES

(1) Moharana, S.; West, G.; Walker, M.; Yan, X. S.; Loveridge, M. Controlling Li Dendritic Growth in Graphite Anodes by Potassium Electrolyte Additives for Li-Ion Batteries. *ACS Appl Mater Interfaces* **2022**, *14* (37), 42078–42092. https://doi.org/10.1021/acsami.2c11175.

(2) Xu, K. Whether EC and PC Differ in Interphasial Chemistry on Graphitic Anode and How. *J Electrochem Soc* **2009**, *156* (9), A751. https://doi.org/10.1149/1.3166182.

(3) Chung, G.-C.; Kim, H.-J.; Yu, S.-I.; Jun, S.-H.; Choi, J.; Kim, M.-H. Origin of Graphite Exfoliation An Investigation of the Important Role of Solvent Cointercalation. *J Electrochem Soc* **2000**, *147* (12), 4391. https://doi.org/10.1149/1.1394076.

(4) Xiang, H.; Mei, D.; Yan, P.; Bhattacharya, P.; Burton, S. D.; Von Wald Cresce, A.; Cao, R.; Engelhard, M. H.; Bowden, M. E.; Zhu, Z.; Polzin, B. J.; Wang, C. M.; Xu, K.; Zhang, J. G.; Xu, W. The Role of Cesium Cation in Controlling Interphasial Chemistry on Graphite Anode in Propylene Carbonate-Rich Electrolytes. *ACS Appl Mater Interfaces* **2015**, *7* (37), 20687–20695. https://doi.org/10.1021/acsami.5b05552.

(5) Li, Q.; Lu, D.; Zheng, J.; Jiao, S.; Luo, L.; Wang, C. M.; Xu, K.; Zhang, J. G.; Xu, W. Li+-Desolvation Dictating Lithium-Ion Battery's Low-Temperature Performances. *ACS Appl Mater Interfaces* **2017**, *9* (49), 42761–42768. https://doi.org/10.1021/acsami.7b13887.

(6) Spahr, M. E.; Palladino, T.; Wilhelm, H.; Würsig, A.; Goers, D.; Buqa, H.; Holzapfel, M.; Novák, P. Exfoliation of Graphite during Electrochemical Lithium Insertion in

Ethylene Carbonate-Containing Electrolytes. *J Electrochem Soc* **2004**, *151* (9), A1383. https://doi.org/10.1149/1.1775224.

(7) Zhao, H.; Park, S.-J.; Shi, F.; Fu, Y.; Battaglia, V.; Ross, P. N.; Liu, G. Propylene Carbonate (PC)-Based Electrolytes with High Coulombic Efficiency for Lithium-Ion Batteries. *J Electrochem Soc* **2014**, *161* (1), A194–A200. https://doi.org/10.1149/2.095401jes.

(8) Hosaka, T.; Kubota, K.; Hameed, A. S.; Komaba, S. Research Development on K-Ion Batteries. *Chemical Reviews*. 2020, pp 6358–6466. https://doi.org/10.1021/acs.chemrev.9b00463.

(9) Okoshi, M.; Yamada, Y.; Komaba, S.; Yamada, A.; Nakai, H. Theoretical Analysis of Interactions between Potassium Ions and Organic Electrolyte Solvents: A Comparison with Lithium, Sodium, and Magnesium Ions. *J Electrochem Soc* **2017**, *164* (2), A54–A60. https://doi.org/10.1149/2.0211702jes.

(10) Biesinger, M. C. *X-Ray Photoelectron Spectroscopy Reference Pages*. http://www.xpsfitting.com/2020/02/potassium.html.

(11) Garner, E. L.; Murphy, T. J.; Gramlich, J. W.; Paulsen, P. J.; Barnes, I. L. Absolute Isotopic Abundance Ratios and the Atomic Weight of a Reference Sample of Potassium. *J Res Natl Bur Stand Sect A Phys Chem* **1975**, *79 A* (6), 713–725. https://doi.org/10.6028/jres.079A.028.
